# Learning Curve of Transperineal MRI/US Fusion Prostate Biopsy: 4-Year Experience

**DOI:** 10.3390/life13030638

**Published:** 2023-02-24

**Authors:** Po-Fan Hsieh, Po-I Li, Wei-Ching Lin, Han Chang, Chao-Hsiang Chang, Hsi-Chin Wu, Yi-Huei Chang, Yu-De Wang, Wen-Chin Huang, Chi-Ping Huang

**Affiliations:** 1 Department of Urology, China Medical University Hospital, Taichung 404, Taiwan; 2 School of Medicine, China Medical University, Taichung 404, Taiwan; 3 Department of Radiology, China Medical University Hospital, Taichung 404, Taiwan; 4 Department of Pathology, China Medical University Hospital, Taichung 404, Taiwan; 5 Department of Urology, China Medical University Beigang Hospital, Yunlin 651, Taiwan; 6 Graduate Institute of Biomedical Sciences, School of Medicine, China Medical University, Taichung 404, Taiwan; 7 International Master’s Program of Biomedical Sciences, School of Medicine, China Medical University, Taichung 404, Taiwan

**Keywords:** learning curve, MRI/US fusion prostate biopsy, transperineal biopsy, Taiwan

## Abstract

This study aimed to evaluate the learning curve of transperineal magnetic resonance imaging (MRI)/ultrasound (US) fusion biopsy in a team composed of a single surgeon, a single radiologist, and a single pathologist. We prospectively enrolled 206 patients undergoing MRI/US fusion prostate biopsy and divided them into four cohorts by the year of biopsy. We analyzed temporal changes in clinically significant prostate cancer (csPC) detection rate, percentage of positive cores on biopsy, and Gleason upgrading rate after radical prostatectomy. The csPC detection rate by MRI/US fusion targeted biopsy (TB) increased significantly (from 35.3% to 60.0%, *p* = 0.01). With increased experience, the csPC detection rates for small (≤1 cm) and anterior target lesions gradually increased (from 41.2% to 51.6%, *p* = 0.5; from 54.5% to 88.2%, *p* = 0.8, respectively). The percentage of positive cores on TB increased significantly (from 18.4% to 44.2%, *p* = 0.001). The Gleason upgrading rate gradually decreased (from 22.2% to 11.1%, *p* = 0.4). In conclusion, with accumulated experience and teamwork, the csPC detection rate by TB significantly increased. Multidisciplinary team meetings and a free-hand biopsy technique were the key factors for overcoming the learning curve.

## 1. Introduction

Prostate cancer is the most prevalent noncutaneous malignancy, and it is also the second leading cause of cancer death in males [1]. Even in men with a benign initial TURP (transurethral resection of the prostate), the 10-year accumulated incidence of prostate cancer was 4.0% [2,3]. In Taiwan, with the increasing use of prostate-specific antigen (PSA) screening, the incidence of prostate cancer has increased over the past decades [4]. Traditionally, prostate cancer has been diagnosed using a sextant or systematic biopsy (SB), which may miss 20% to 30% of clinically significant prostate cancer (csPC) because of the random sampling approach [5,6]. Recently, Yang et al. reported that in 295 men with benign preoperative biopsies undergoing TURP or simple prostatectomy, 57 (19%) were found to have prostate cancer incidentally [7]. With technical advances in multiparametric magnetic resonance imaging (mpMRI) and promotion by the European Society of Urogenital Radiology and American College of Radiology in the past decades, mpMRI has become an important imaging tool in the diagnostic pathway of prostate cancer [8]. It can help detect more csPC and less clinically insignificant prostate cancer compared with transrectal ultrasound (US)-guided SB, both in biopsy-naïve men and men with prior negative biopsies [9,10,11]. In addition, up to one third of unnecessary biopsy procedures can be avoided [10,12]. Eklund et al. evaluated the role of MRI in the screening of prostate cancer in a large population-based study in Sweden. They found that using MRI for the selection and guidance of prostate biopsy yielded a noninferior detection rate for csPC (21% vs. 18%, *p* < 0.001 for noninferiority), and that it detected less clinically insignificant prostate cancer (4% vs. 12%; −8 percentage points of difference; 95% CI, −11 to −5) compared with standard biopsy [13]. Currently, the European Association of Urology (EAU), American Urological Association (AUA), National Institute for Health and Clinical Excellence (NICE), and National Comprehensive Cancer Network (NCCN) guidelines all strongly recommended mpMRI prior to prostate biopsy [14,15,16,17].

The application of mpMRI for prostate biopsy includes MRI in-bore biopsy, cognitive or visual-estimation biopsy, and MRI/US or software fusion biopsy [18]. Theoretically, MRI/US fusion biopsy could provide more objective and precise lesion targeting than cognitive biopsy, and it was also more cost-effective than MRI in-bore biopsy. MRI/US fusion works by overlying segmented MR images on real-time US, enabling the operators to sample the target lesions accurately and efficiently. Various commercial MRI/US fusion platforms are available, and they differ in the methods of US image acquisition, tracking mechanism, and methods of image registration [19]. However, MRI/US fusion prostate biopsy is not yet popular worldwide because of the technical complexity in software management, as well as interpretation of mpMRI and US. Therefore, the issue of learning curve in MRI/US fusion prostate biopsy is important. A detailed evaluation of the learning curve can help novice urologists to become familiar with the techniques of MRI/US fusion biopsy more quickly.

The sampling of prostatic tissue can be done through a transrectal biopsy or transperineal biopsy. In the setting of SB, no significant difference in cancer detection rate has been reported between transrectal biopsy and transperineal biopsy, both in observational studies and randomized controlled trials [20]. In the era of MRI, some studies have reported higher cancer detection rates with transperineal MRI/US fusion biopsy compared with transrectal fusion biopsy [21,22]. In a recent large multicenter cohort study, Zattoni et al. reported that transperineal fusion biopsy detected more csPC than transrectal fusion biopsy (49% vs. 35%, *p* < 0.01), especially when the cancer was located in the apex, transition zone, central zone, or anterior zone [23]. Transperineal MRI/US fusion biopsy tended to take a little more procedural time than transrectal fusion biopsy [24]. Regarding post-biopsy complications, in a comparative study of prospective databases, Hsieh et al. found a higher rate of postoperative urinary retention in men undergoing transperineal fusion biopsy compared with transrectal fusion biopsy (18.5% vs. 4.7%, *p* = 0.009), but no individuals who underwent transperineal fusion biopsy developed sepsis [22]. Hence, the latest EAU guidelines recommend that a transperineal route should be the first choice of prostate biopsy due to minimal infectious complications and a possibly superior cancer detection rate compared with a transrectal route [14]. Nevertheless, most studies on the learning curve of MRI/US fusion prostate biopsy have focused on transrectal biopsy, and relatively few studies have reported on the learning curve of transperineal MRI/US fusion biopsy [24,25].

Since current international guidelines strongly recommend mpMRI before prostate biopsy and a transperineal approach for tissue sampling, we conducted this prospective study to evaluate and analyze the learning curve of transperineal MRI/US fusion prostate biopsy in a team consisting of a single surgeon, a single radiologist, and a single pathologist. The aim of this study was to help urologists to shorten the learning curve of transperineal MRI/US fusion biopsy.

## 2. Materials and Methods

### 2.1. Study Population

After approval by the Research Ethics Committee of China Medical University Hospital, Taichung, Taiwan (protocol number: CMUH109-REC1-045), we prospectively collected data of patients undergoing transperineal MRI/US fusion prostate biopsy from May 2019 to September 2022 at a tertiary referral center. The inclusion criteria were men with a serum PSA level ≥ 4 ng/mL or abnormal digital rectal examination. Prebiopsy mpMRI with a PI-RADS score of ≥3 was required. Men with a history of prostate cancer, bacterial prostatitis within 3 months, or an inability to sign informed consent were excluded from this study. After obtaining informed consent, we recorded data on clinical characteristics including age, serum PSA level, digital rectal examination findings, status of biopsy-naïve or previous negative biopsy, prostate volume, number, location, and PI-RADS score of the target lesion, number of biopsy cores, as well as histological results. For biopsy-proven prostate cancer patients who underwent robotic-assisted radical prostatectomy, pathological reports were also collected.

### 2.2. MRI Protocol

All mpMRI scans were performed using a 3-T scanner (Signa HDxt, GE Healthcare, Milwaukee, WI, USA) with an eight-channel high resolution cardiac array coil. No endorectal coil was used. The scanning protocol was performed as described previously [26]. In brief, the protocol included T2-weighted imaging to provide anatomical information and diffusion-weighted imaging with *b* values of 0–1000 s/mm^2^, apparent diffusion coefficient map, and dynamic contrast-enhanced imaging as functional imaging modalities (Supplementary Table S1). All mpMRI scans were interpreted by a radiologist (W.C.L.) with more than 10 years of experience in reading prostate mpMRI. Each suspicious lesion was scored in accordance with the Prostate Imaging-Reporting and Data System v2.1 [8]. Before biopsy, one urologist (P.F.H.) reviewed the mpMRI and identified suspicious lesions with a PI-RADS score ≥ 3 as the target lesions. The index lesion was defined as the target lesion with the highest PI-RADS score. If there were two or more target lesions with the same PI-RADS score, the index lesion was defined as the largest one. T2-weighted imaging was used for contouring the prostate and target lesions, and then a 3-D model of the prostate and target lesions was built using a BioJet (D&K Technologies GmbH, Barum, Germany) or bkFusion (BK Medical, Herlev, Denmark) system.

### 2.3. Biopsy Protocol

**Figure 1:**
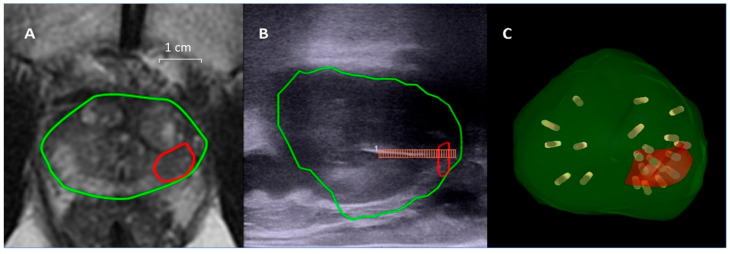
Example of MRI/US fusion prostate biopsy. A target lesion of 10 mm (red circle) was noted in the left peripheral zone near the apex of the prostate (green circle), and the PI-RADS score was 4 (**A**). The sagittal view of real-time US showed the biopsy needle puncturing through the target (**B**). All of the biopsy trajectories were recorded in a 3D model (**C**). Gleason 3 + 4 adenocarcinoma involving 80% of the TB cores was found on histopathological analysis. The patient received radical prostatectomy, and Gleason 3 + 4 adenocarcinoma on the left lobe was confirmed, involving 1.6% of the prostate volume.

Under general anesthesia with endotracheal intubation and prophylactic antibiotics with levofloxacin, the patients were placed in the lithotomy position. A transrectal probe (BK 8848, BK Medical, Peabody, MA, USA) was first used to obtain a US scan of the prostate. The segmented mpMRI images were then overlaid on the real-time US images in the fusion platform using a rigid or elastic registration. With regards to the image tracking mechanism, the BioJet system used mechanical arms with built-in encoders, and the bkFusion system used electromagnetic navigation. After confirming the alignment of prostate contours on mpMRI and US in both the transverse view and sagittal view, MRI/US fusion targeted biopsy (TB) was done with at least two cores in each target lesion. Subsequently, SB was performed following the Ginsburg protocol, in which biopsy cores were taken from 12 sectors of the prostate [27]. The biopsy samples were obtained using an 18 G biopsy gun with a specimen size of 22 mm (Bard Magnum; Bard Medical, Covington, KY, USA). The SB samples were put in 12 bottles according to each sector, and the TB samples were put in additional bottles according to the number of target lesions. All biopsies were performed transperineally by a single urologist (P.F.H.) with 7 years of experience in transrectal cognitive prostate biopsy. Initially a brachytherapy grid was fixed on the stepper on the mechanical arm in the BioJet system to guide the biopsy routes, and since 2021 a free-hand biopsy technique without the brachytherapy grid has been used exclusively [28,29]. Only a free-hand biopsy technique was allowed in the bkFusion system. All of the biopsy trajectories were recorded on the MRI/US fusion platform, and a video of the biopsy procedure was recorded for each patient (Figure 1).

### 2.4. Histopathological Analysis

One pathologist (H.C.) with more than 20 years of experience interpreted all prostate biopsy specimens. Prostate cancer was graded in accordance with the 2014 International Society of Urological Pathology Consensus Conference guidelines [30]. The specimen length of every biopsy core and the percentage of cancer involvement were measured. We defined csPC as prostate cancer with a Gleason score ≥ 3 + 4 or Gleason grade group ≥ 2. A multidisciplinary team meeting with the pathologist, urologist, and radiologist was held every 2 weeks, during which the procedural videos, imaging details on mpMRI, biopsy trajectories recorded on US and mpMRI, and the cancer grade group, as well as cell architecture on histopathology in each case, were reviewed. For the prostate cancer patients who underwent robotic-assisted radical prostatectomy, we compared the Gleason grade group between biopsy specimens and radical prostatectomy pathology. Gleason upgrading was defined as a higher Gleason grade group detected from the radical prostatectomy pathology than the Gleason grade group detected from the combination of TB and SB. We also checked the concordance of the index lesion between mpMRI and radical prostatectomy pathology [31].

### 2.5. Outcome Measures and Statistical Analysis

Continuous variables were reported as means (standard deviation, SD), and categorical variables were reported as proportions. The study population was divided into four cohorts by the year of biopsy (2019, 2020, 2021, and 2022, respectively). We analyzed the temporal changes in csPC detection rate according to the different biopsy methods (TB, SB, and combination of TB and SB). We then separated the study population into those with a PI-RADS score ≥ 4 and those with a PI-RADS score of 3, and the temporal changes in csPC detection rate in each group were assessed. In addition, we analyzed the temporal changes in csPC detection rate in patients with target lesions ≤ 1 cm and in those with target lesions in the anterior lobe. Logistic regression analysis was performed to evaluate possible predictors for csPC. We also analyzed the temporal changes in the percentage of positive cores on TB and SB. Finally, in biopsy-proven prostate cancer patients, we assessed the Gleason upgrading rate after robotic-assisted radical prostatectomy over the 4 years. Continuous and categorical variables were compared using one-way ANOVA and the Kruskal–Wallis test, respectively. The temporal changes in csPC detection rate, percentage of positive cores on TB/SB, and Gleason upgrading rate were analyzed using the Cochran-Armitage trend test. The csPC detection rates by TB, SB, and a combination of TB and SB were compared using McNemar’s test in each year. All statistical analyses were performed using SPSS version 22 (IBM Corp., Armonk, NY, USA), assuming a two-sided test with an alpha of 5% for statistical significance.

## 3. Results

A total of 206 patients with 311 target lesions were enrolled in this study. Most of the transperineal MRI/US fusion biopsies were done using the BioJet system, and only four cases were done using the bkFusion system. The mean age of the patients was 67.3 (SD 8.8, range 42–90) years. The mean serum PSA level was 10.3 ng/mL (SD 9.8, range 0.55–86.1) ng/mL, and 151 (73.3%) patients were biopsy naïve. The PI-RADS scores of the index lesions were 3, 4, and 5 in 45 (21.8%), 98 (47.6%), and 63 (30.6%) patients, respectively.

The study population was divided into four cohorts according to the year of biopsy. The baseline characteristics, including age, serum PSA level, prostate volume, size of the index lesion, proportion of abnormal digital rectal examinations, and distribution of PI-RADS scores, were comparable among the four cohorts. The number of biopsy cores per target was highest in 2020 (up to a mean of 7.1 cores) and then decreased gradually. The proportion of negative biopsies within 5 years was lowest in 2022 (only 15.7%, Table 1). Overall, 128 (62.1%) patients were diagnosed with prostate cancer, and 117 (56.8%) patients were diagnosed with csPC. The proportions of csPC were 28.9%, 51.0%, and 85.7% in the patients who had an index lesion with a PI-RADS score of 3, 4, and 5, respectively.

**Figure 2:**
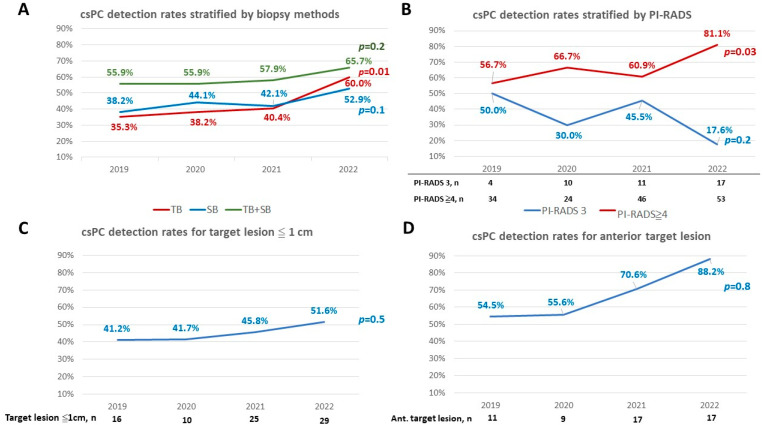
The csPC detection rates in different biopsy methods, PI-RADS scores, target lesions ≤ 1 cm, and anterior target lesions. The csPC detection rates by TB alone improved with time (from 35.3% in 2019 to 60.0% in 2022, *p* = 0.01). The combination of TB and SB yielded the highest csPC detection rates all over the four years (**A**). The csPC detection rates for PI-RADS score ≥ 4 lesions increased significantly with time (from 56.7% in 2019 to 81.1% in 2022, *p* = 0.03, (**B**)). The csPC detection rates for target lesions ≤ 1 cm had a non-statistically significant increase with time (from 41.2% in 2019 to 51.6% in 2022, *p* = 0.5, (**C**)). The csPC detection rates for target lesions in the anterior lobe had a non-statistically significant increase with time (from 54.5% in 2019 to 88.2% in 2022, *p* = 0.8, (**D**)).

The csPC detection rate by TB increased significantly with time (from 35.3% in 2019 to 60.0% in 2022, *p* = 0.01), while the csPC detection rate by SB did not increase significantly with time (from 38.2% in 2019 to 52.9% in 2022, *p* = 0.1). In the first 3 years, SB non-significantly detected more csPC than TB (38.2% vs. 35.3%, 44.1% vs. 38.2%, and 42.1% vs. 40.4%; *p* = 1, 0.6, and 1, respectively). However, in the fourth year, the detection rate of csPC on TB outweighed that on SB non-significantly (60.0% vs. 52.9%, *p* = 0.18). Notably, the combination of TB and SB yielded the highest csPC detection rates in all 4 years. Specifically, the csPC detection rates with the combination of TB and SB were higher than TB alone (55.9% vs. 35.3%, 55.9% vs. 38.2%, 57.9% vs. 40.4%; *p* = 0.01, 0.03, 0.002, respectively) in the first 3 years. In the fourth year, the csPC detection rate was similar between TB alone and the combination of TB and SB (60.0% vs. 65.7%, *p* = 0.1, Figure 2A). Furthermore, the csPC detection rate for lesions with a PI-RADS score ≥ 4 increased significantly with time (from 56.7% in 2019 to 81.1% in 2022, *p* = 0.03), whereas the csPC detection rate for lesions with a PI-RADS score of 3 did not increase with time (from 50.0% in 2019 to 17.6% in 2022, *p* = 0.2, Figure 2B). There were 80 small (≤1 cm) target lesions and 54 target lesions in the anterior lobe, respectively. The csPC detection rate for target lesions ≤ 1 cm increased with time, although not significantly (from 41.2% in 2019 to 51.6% in 2022, *p* = 0.5, Figure 2C). The csPC detection rate for target lesions in the anterior lobe also increased with time, but was also without statistical significance (from 54.5% in 2019 to 88.2% in 2022, *p* = 0.8, Figure 2D).

In univariate logistic regression analysis, we found age (OR 1.10, 95% CI 0.86–1.42), PSA (OR 1.12, 95% CI 1.06–1.18), abnormal DRE (OR 1.99, 95% CI 1.05–3.75), prostate volume (OR 0.96, 95% CI 0.95–0.98), biopsy cores per target (OR 1.25, 95% CI 1.08–1.45), as well as the size (OR 1.10, 95% CI 1.05–1.15) and PI-RADS score (OR 3.82, 95% CI 2.39–6.12) of the index lesion were associated with the diagnosis of csPC. In multivariate logistic regression analysis, the year of biopsy (OR 1.51, 95% CI 1.03–2.20), age (OR 1.13, 95% CI 1.07–1.20), PSA (OR 1.12, 95% CI 1.03–1.22), prostate volume (OR 0.95, 95% CI 0.93–0.97), and PI-RADS score of the index lesion (OR 2.38, 95% CI 1.16–4.89) were significant predictors for csPC (Table 2).

**Figure 3:**
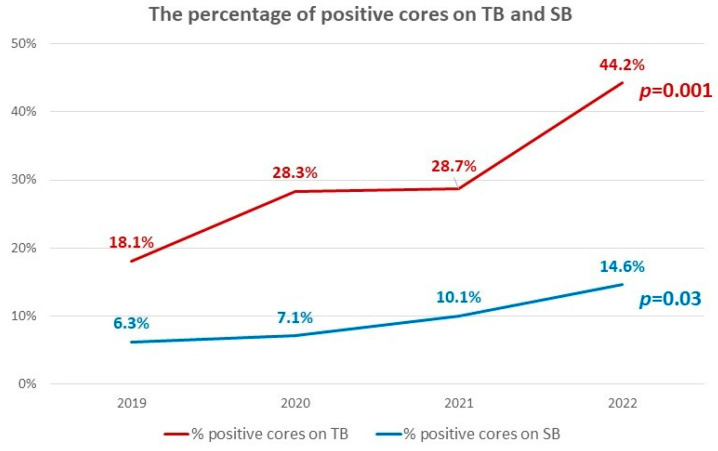
The percentage of positive cores on TB and SB. There were significant increases of the percentage of positive cores on TB and SB with time (from 18.1% in 2019 to 44.2% in 2022 for TB, *p* = 0.001; from 6.3% in 2019 to 14.6% in 2022 for SB, *p* = 0.03).

The percentage of positive cores on TB increased significantly with time (from 18.1% in 2019 to 44.2% in 2022, *p* = 0.001). The percentage of positive cores on SB also increased significantly with time (from 6.3% in 2019 to 14.6% in 2022, *p* = 0.03). The percentage of positive cores on TB was higher than that on SB in each year (18.1% vs. 6.3%, 28.3% vs. 7.1%, 28.7% vs. 10.1%, and 44.2% vs. 14.6% in 2019, 2020, 2021, and 2022; *p* = 0.04, 0.01, 0.02, and <0.001, respectively, Figure 3).

**Figure 4:**
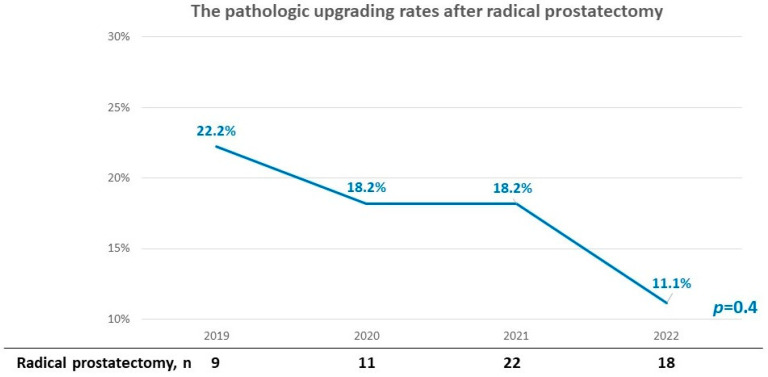
The pathologic upgrading rates after radical prostatectomy. Gleason upgrading rates had a non-statistically significant decrease with time (from 22.2% in 2019 to 11.1% in 2022, *p* = 0.4).

In the 60 prostate cancer patients who underwent robotic-assisted radical prostatectomy, the index lesions on mpMRI were all concordant with the index lesion on radical prostatectomy pathology. In addition, the Gleason upgrading rate decreased with time, although without significance (from 22.2% in 2019 to 11.1% in 2022, *p* = 0.4, Figure 4).

## 4. Discussion

In summary, our prospective study showed that with close collaboration between a urologist, radiologist, and pathologist, the csPC detection rate by transperineal MRI/US fusion TB of the prostate increased significantly over the 4-year study period (from 35.3% to 60.0%, *p* = 0.01). Combining TB and SB yielded the highest csPC detection rate in each year. With increased experience, the csPC detection rates for small (≤1 cm) and anterior target lesions gradually increased (from 41.2% to 51.6%, *p* = 0.5 and from 54.5% to 88.2%, *p* = 0.8, respectively), and the percentage of positive cores on TB increased significantly (from 18.1% to 44.2%, *p* = 0.001). In addition, the Gleason upgrading rates after radical prostatectomy gradually decreased (from 22.2% to 11.1%, *p* = 0.4).

Gaziev et al. conducted the first prospective study to evaluate the learning curve of transperineal MRI/US fusion prostate biopsy [25]. They enrolled a total of 340 men and divided them into five groups representing consecutive time intervals and found that the prostate cancer detection rate increased by 36% when comparing the first and last groups. However, the sequences of MRI included only T2-weighted imaging and diffusion-weighted imaging. The lack of dynamic contrast-enhanced imaging was inconsistent with the current PI-RADS guidelines, and it may have decreased the sensitivity in detecting csPC [8,32]. In addition, the MRI was read by two radiologists, and biopsy procedures were done by three urologists. Inter-reader and inter-operator variability may have influenced evaluation of the learning curve. Subsequently, Halstuch et al. conducted a study to characterize the learning curve of transperineal MRI/US fusion prostate biopsy by a single surgeon [24]. They found that at least 125 procedures were needed to achieve proficiency in transperineal MRI/US fusion biopsy. However, the MRI was interpreted by more than 10 radiologists, and more than 60% of the suspicious lesions on MRI had a PI-RADS score of only 3, implying possible inexperience in the interpretation of MRI. In contrast, our study is the first prospective study in which MRI/US fusion biopsy outcomes were obtained by only one surgeon and one radiologist, and this may better illustrate the learning curve of transperineal MRI/US fusion biopsy.

In our study, the significantly improved csPC detection rate from 35.3% to 60.0% by TB over the 4-year study period may be attributed to the following reasons. First, the learning curve for performing transperineal MRI/US fusion prostate biopsy was overcome, and the key factor may have been the biweekly multidisciplinary team meetings. By reviewing the histological reports, MRI findings, biopsy trajectories, and procedural videos, we could thoroughly assess the false-negative and false-positive findings on mpMRI. This allowed the radiologist to gain experience in MRI interpretation and lesion contouring. In addition, the urologist could also learn how to avoid targeting and registration errors. Second, we have used a free-hand biopsy technique since 2021, and the csPC detection rate by TB outweighed that by SB in 2022, albeit without significance (60.0% vs. 52.9%, *p* = 0.18). During transperineal prostate biopsy, the anterior lobe or anterior lateral horn may be located behind the pubic symphysis or pubic ramus, and it is difficult to approach these areas using a brachytherapy grid. Instead, using a free-hand biopsy technique, we could puncture the perineal skin at any site through any angle and place the biopsy needle at any location in the prostate. Therefore, a free-hand biopsy technique could facilitate sampling of anterior and anterolateral target lesions more easily. Previous studies have also shown that a free-hand biopsy technique can detect csPC with a lower number of biopsy cores, reduce sampling of the transition zone, shorten the biopsy procedure time, and result in a lower complication rate [28,29]. Our study demonstrated a non-significant improvement in anterior csPC detection rate (from 54.5% to 88.2%, *p* = 0.8) over the 4-year study period. Accumulated experience with the free-hand biopsy technique may allow for continued improvement in the anterior csPC detection rate.

Another important finding of this study is that the mean percentage of positive cores on TB increased from 18.1% to 44.2% over the 4-year study period. In a prospective study of 209 patients undergoing transrectal MRI/US fusion biopsy, Cata et al. reported that the median percentage of positive cores on TB increased from 0% in the first 52 patients to 66.7% in the last 53 patients (*p* = 0.06) [33]. In addition, Kasabwala et al. retrospectively analyzed the accuracy and histological quality of MRI/US fusion biopsy in 173 patients and reported that the mean distance from biopsy trajectory to target decreased from 6.7 mm in tertile 1 to 0.06 mm in tertile 3 (*p* < 0.01). The amount of fibromuscular tissue and number of cores missing the prostate also decreased significantly over time [34]. Taken together, these quantitative analyses showed that overcoming the learning curve of MRI/US fusion biopsy could reduce targeting errors.

Through accurate targeting, MRI/US fusion prostate biopsy should theoretically be able to detect even small lesions. A previous meta-analysis found no significant advantage of MRI/US fusion compared with visual estimation in the detection of overall prostate cancer or csPC for experienced operators [18]. Wysock et al. compared the accuracy of MRI/US fusion and visual estimation in a prospective study and showed that MRI/US fusion performed better than visual estimation for smaller target lesions [35]. Checcucci et al. also reported that for target lesions < 8 mm, the cancer detection rate increased significantly with operator experience [36]. In particular, experience of at least 100 biopsy procedures was needed to correctly sample these small lesions. They suggested that patients with small target lesions should be managed at referral centers with a high number of procedures per year. In our study there were only 80 small (≤ 1 cm) target lesions, and we showed that the csPC detection rate gradually increased over the 4-year study period (from 41.2% to 51.6%, *p* = 0.5). Due to the limited number of cases, we did not observe a statistically significant improvement in the csPC detection rate. Further large-scale studies are warranted to investigate the impact of MRI/US fusion on the detection of small target lesions.

Another important finding of our study is the value of combining TB and SB. Our results showed that the combination of TB and SB always yielded the highest csPC detection rate compared with TB or SB alone. In the first 3 years, the slightly lower csPC detection rate by TB than SB may have been due to inexperience with the biopsy technique or image interpretation at the beginning of the learning curve. The addition of SB to TB, especially sampling around the target lesions, could help overcome targeting and registration errors [37]. In the fourth year, although the csPC detection rate by TB significantly improved, SB still played a role in the detection of some lesions that were not seen on MRI, such as small but high-grade cancer, cancer with a heterogeneous morphology, or cancer with cribriform architecture [38,39]. As a result, SB remains indispensable in the era of TB in terms of maximizing the cancer detection rate. More importantly, focal therapy has emerged as a new treatment modality for localized prostate cancer, and SB could provide detailed preoperative mapping. In summary, transperineal SB can help to identify clinically significant but unsuspected prostate cancer outside the target lesion for ablation. This may help urologists to be more confident in selecting appropriate patients for focal therapy and achieve adequate cancer control whilst preserving as much normal prostatic tissue as possible [40,41].

The combination of mpMRI and serum or urinary biomarkers has gained attention in the diagnosis of prostate cancer in recent years. For example, integrating PCA3, 4Kscore, ExosomeDx, and mpMRI in the diagnostic pathway could help reduce unnecessary biopsies and the detection of indolent cancer [42]. The combination of mpMRI and prostate health index (PHI) has also been shown to improve the predictive value. Hsieh et al. reported that restricting biopsy to men with PI-RADS 5 lesions and PI-RADS 3/4 lesions plus PHI ≥ 30 could avoid 50% of biopsies and miss only 4.2% of csPC [26]. In addition, Fan et al. reported that PHI had a higher predictive power for PI-RADS 3 lesions than PI-RADS 4/5 lesions (AUC 0.884 and 0.792, respectively) [43]. Moreover, the combination of mpMRI and PHI could help predict extraprostatic extension after radical prostatectomy, as well as the histological tumor diameter [31,44]. Extensive research is currently ongoing to evaluate the role of molecular biomarkers in conjunction with mpMRI [45].

The final important finding of this study is that the Gleason upgrading rate decreased over the 4-year study period, albeit without significance (from 22.2% in 2019 to 11.1% in 2022, *p* = 0.4). Only 60 patients received radical prostatectomy in this study, so we were not able to demonstrate a significant correlation between the learning curve and temporal decrease in Gleason upgrading. Calio et al. conducted a study of 1528 patients to assess the learning curve of transrectal MRI/US fusion prostate biopsy [46], and divided the study population into three cohorts by the time of biopsy. They also found that TB was associated with a non-significant decrease in Gleason upgrading rate (40.0%, 32.3%, and 29.5% in cohort 1, 2, and 3, respectively; *p* = 0.428). However, there was a significant decrease in risk category upgrading rate (28.9%, 16.1%, and 10.1% in cohort 1, 2, and 3, respectively; *p* = 0.01). The relatively lower Gleason upgrading rate in our study may be because we sampled more biopsy cores, and a nearly transperineal template mapping biopsy may be able to assess the Gleason grade group more correctly [47].

There were some limitations to this study. First, the urologist who performed the transperineal MRI/US fusion prostate biopsies already had some experience of transrectal cognitive biopsy, and the radiologist had more than 10 years of experience in reading mpMRI. This prior experience could have shortened the learning curve of MRI/US fusion biopsy [22,48]. Thus, our findings may not be extrapolated to other hospitals where mpMRI and MRI/US fusion prostate biopsy have recently been introduced. However, our results showed that the importance of regular multidisciplinary team meetings cannot be overemphasized, and that a free-hand biopsy technique could further increase the cancer detection rate. We believe that our experience could help novice urologists or radiologists to become proficient with transperineal MRI/US fusion prostate biopsy more quickly. Furthermore, the rapid development in machine learning in prostate MRI may aid in PI-RADS categorization, image segmentation, and coregistration, and may also shorten the learning curve for MRI/US fusion biopsy in the future [49,50]. Second, most of our cases were performed using the BioJet system, and thus our outcomes may not be totally applicable to other MRI/US fusion platforms which use other mechanisms of image tracking or guidance of biopsy trajectories. Third, although all MRI were read by a single radiologist and all biopsy procedures were performed by a single urologist, we could not separate the learning curve into improved MRI interpretation, image registration, or lesion targeting during biopsy. In other words, the learning curve reflected the outcome of teamwork among the urologist, radiologist, and pathologist, reinforcing the necessity of multidisciplinary team meetings. Finally, the csPC detection rate did not reach a plateau by the end of this study. Therefore, we could not draw a definite conclusion about how many cases are needed to completely overcome the learning curve of transperineal MRI/US fusion prostate biopsy. We hope to include more patients in future studies.

## 5. Conclusions

This study is the first prospective study to evaluate the learning curve of transperineal MRI/US fusion prostate biopsy performed by a single surgeon, single radiologist, and single pathologist. With accumulated experience and collaborative teamwork, the csPC detection rate by TB significantly increased. Multidisciplinary team meetings and a free-hand biopsy technique were the key factors for overcoming the learning curve.

## Figures and Tables

**Table 1:** Demographic characteristics of the study population.

Year	2019	2020	2021	2022	*p* Value
Case no.	35	36	59	76	
Age, mean ± SD	67.1 ± 8.3	64.6 ± 9.0	67.8 ± 8.6	67.5 ± 8.0	0.17
PSA (ng/mL), mean ± SD	9.9 ± 6.5	11.9 ± 13.6	10.0 ± 11.5	11.6 ± 11.0	0.76
Prostate volume (cm^3^), mean ± SD	46.5 ± 34.6	48.5 ± 25.7	52.2 ± 24.5	52.8 ± 24.9	0.65
Index lesion size (cm), mean ± SD	14.3 ± 8.6	15.2 ± 8.2	12.5 ± 6.5	14.0 ± 9.2	0.4
Biopsy cores per target (n), mean ± SD	5.0 ± 2.1	7.1 ± 2.3	6.1 ± 2.3	5.3 ± 1.4	0.01
Systematic biopsy cores (n), mean ± SD	18.3 ± 3.7	19.9 ± 3.9	19.2 ± 3.1	18.1 ± 2.9	0.09
PI-RADS score of index lesion					0.32
3	4	10	12	19	
4	17	13	31	37	
5	14	13	16	20	
Negative biopsy within 5 years, n (%)	9 (25.7%)	11 (30.6%)	23 (39.0%)	11 (14.5%)	0.02
Abnormal DRE, n (%)	11 (31.4%)	8 (22.2%)	19 (32.2%)	18 (23.7%)	0.67

DRE: digital rectal examination, SD: standard deviation, PI-RADS: Prostate Imaging-Reporting and Data System, PSA: prostate specific antigen.

**Table 2:** Logistic regression analysis for csPC detection.

	Univariate			Multivariate		
	OR	95% CI	*p* Value	OR	95% CI	*p* Value
Year of biopsy	1.10	0.86–1.42	0.4	1.51	1.03–2.20	0.03
Age	1.10	1.06–1.14	<0.001	1.13	1.07–1.20	<0.001
PSA	1.12	1.06–1.18	<0.001	1.12	1.03–1.22	0.008
Prostate volume	0.96	0.95–0.98	<0.001	0.95	0.93–0.97	<0.001
Size of Index lesion	1.10	1.05–1.15	<0.001	1.02	0.95–1.09	0.6
Biopsy cores per target	1.25	1.08–1.45	0.003	1.10	0.89–1.36	0.4
PI-RADS score of index lesion	3.82	2.39–6.12	<0.001	2.38	1.16–4.89	0.02
Negative biopsy within 5 years	0.73	0.39–1.36	0.32	0.50	0.19–1.27	0.14
Abnormal DRE	1.99	1.05–3.75	0.03	1.05	0.43–2.57	0.92

CI: confidence interval, OR: odds ratio.

## Data Availability

Data were contained within the article.

## Supplementary Materials

The following supporting information can be downloaded at: https://www.mdpi.com/article/10.3390/life13030638/s1, Table S1: Image parameters.
