# Supplementary material for: Learning Curve of Transperineal MRI/US Fusion Prostate Biopsy: 4-Year Experience
Source: Life (Basel). 2023 Feb 24;13(3):638. doi: 10.3390/life13030638 (PMC10059778; doi:10.3390/life13030638)
Supplement: Supplementary file 1 [file life-13-00638-s001.zip › life-2221608-supplementary.pdf]

Supplementary Table S1. Image parameters.

	T2WI	DWI	DCE
Sequence	SE	EP/SE	GR
TE	126	71	1.32
TR	3500	5725	3.708
FOV (cm)	20	20	24
Slice thickness (mm)	3	3	4
Matrix	256x256	100x130	128x128
Time (min'sec)	3'40	6'40	5'50
spacing between slices	4	4	4
Flip angle	90	90	12
B value	-	1000	-
Phase number	-	-	36
